# ARACNe-based inference, using curated microarray data, of *Arabidopsis thaliana* root transcriptional regulatory networks

**DOI:** 10.1186/1471-2229-14-97

**Published:** 2014-04-16

**Authors:** Ricardo A Chávez Montes, Gerardo Coello, Karla L González-Aguilera, Nayelli Marsch-Martínez, Stefan de Folter, Elena R Alvarez-Buylla

**Affiliations:** 1 Laboratorio de Genética Molecular, Desarrollo y Evolución de Plantas, Instituto de Ecología and Centro de Ciencias de la Complejidad (C3), Universidad Nacional Autónoma de México, Ciudad Universitaria, México D.F. 04510, Mexico; 2 Unidad de Cómputo, Instituto de Fisiología Celular, Universidad Nacional Autónoma de México, Ciudad Universitaria, México D.F. 04510, Mexico; 3 Laboratorio Nacional de Genómica para la Biodiversidad (Langebio), Centro de Investigación y de Estudios Avanzados del Instituto Politécnico Nacional, Km 9.6 Libramiento Norte, Carretera Irapuato-León, AP 629, CP 36821 Irapuato, Guanajuato, Mexico; 4 Departamento de Biotecnologıa y Bioquımica, Centro de Investigación y de Estudios Avanzados del Instituto Politécnico Nacional, Km 9.6 Libramiento Norte, Carretera Irapuato-León, AP 629, CP 36821 Irapuato, Guanajuato, Mexico; 5 Present address: Laboratorio Nacional de Genómica para la Biodiversidad (Langebio), Centro de Investigación y de Estudios Avanzados del Instituto Politécnico Nacional, Km 9.6 Libramiento Norte, Carretera Irapuato-León, AP 629, CP 36821 Irapuato, Guanajuato, Mexico

**Keywords:** Transcriptional regulatory networks, Root, Arabidopsis, Transcription factor, ARACNe

## Abstract

**Background:**

Uncovering the complex transcriptional regulatory networks (TRNs) that underlie plant and animal development remains a challenge. However, a vast amount of data from public microarray experiments is available, which can be subject to inference algorithms in order to recover reliable TRN architectures.

**Results:**

In this study we present a simple bioinformatics methodology that uses public, carefully curated microarray data and the mutual information algorithm ARACNe in order to obtain a database of transcriptional interactions. We used data from *Arabidopsis thaliana* root samples to show that the transcriptional regulatory networks derived from this database successfully recover previously identified root transcriptional modules and to propose new transcription factors for the *SHORT ROOT*/*SCARECROW* and *PLETHORA* pathways. We further show that these networks are a powerful tool to integrate and analyze high-throughput expression data, as exemplified by our analysis of a SHORT ROOT induction time-course microarray dataset, and are a reliable source for the prediction of novel root gene functions. In particular, we used our database to predict novel genes involved in root secondary cell-wall synthesis and identified the MADS-box TF *XAL1*/*AGL12* as an unexpected participant in this process.

**Conclusions:**

This study demonstrates that network inference using carefully curated microarray data yields reliable TRN architectures. In contrast to previous efforts to obtain root TRNs, that have focused on particular functional modules or tissues, our root transcriptional interactions provide an overview of the transcriptional pathways present in *Arabidopsis thaliana* roots and will likely yield a plethora of novel hypotheses to be tested experimentally.

## Background

Transcription factors (TFs) play an important role in the regulation of gene expression. The *Arabidopsis thaliana* (Arabidopsis) TF databases Agris [1,2], RARTF [3,4] or DATF [5,6] contain approximately 1900 entries, that correspond to 6.9% of the 27416 protein coding genes present in the TAIR10 genome release. Forward and reverse genetics, inducible expression systems and, more recently, large scale methods, such as chromatin immunoprecipitation followed by array hybridization or massive parallel sequencing, have provided a vast amount of information regarding target genes and functions of many Arabidopsis TFs. However, obtaining a complete overview of the transcriptional interactions for a given organism or developmental process is still a challenging and expensive task. Brady et al. obtained a stele-enriched root TRN containing protein-DNA and protein-protein interactions identified by Y1H and Y2H assays using stele-enriched TFs, the promoters of these same TFs, and promoters from several miRNA coding genes [7]. However, the low percentage of TF promoters bound by at least one TF and the little overlap in expression enrichment between TFs and their targets suggest that several genes that might be important components of the stele TRN could have been missed in this network.

Gene expression microarrays allow for the rapid quantification of the expression level for a large number of genes in a given biological sample. The most used Arabidopsis gene expression microarray is the Affymetrix ATH1-121501 (ATH1) GeneChip microarray. As of October 2010, there were 686 experiments using the ATH1 chip listed in the EBI ArrayExpress database [8]. All of these experiments provide a quantitative analysis of gene expression in Arabidopsis tissues under a variety of experimental conditions and are therefore a suitable data source for Arabidopsis Transcriptional Regulatory Network (TRN) inference. Although databases such as Genevestigator [9,10], ATTED-II [11,12], or BAR Expression Angler [13,14] have tools for the analysis of Arabidopsis microarray data, they either use a limited set of microarray experiments, the AtGenExpress series [15] (ATTED-II and BAR Expression Angler), or their quality controlled, curated, annotated and normalized data is not publicly available (Genevestigator).

In light of these limitations we decided to create our own curated and annotated Arabidopsis microarray database and use this data to infer TRNs. The 686 microarray experiments indexed in the ArrayExpress database contain over 9000 individual chip hybridizations or CEL files. Preliminary work done in our lab showed that network inference from samples obtained from different tissues, for example whole plant, roots and leaves, yields sub-optimal results and the inferred networks are difficult to interpret in a biological context. We therefore decided to use microarray data obtained from a single organ, namely roots. The Arabidopsis root has several characteristics that make it a suitable organ for our purposes: root anatomy is relatively simple and developmental alterations can be readily observed, there is a vast amount of literature regarding root development and root-expressed TFs and, finally, there is a considerable amount of high quality ATH1 microarray data obtained from root samples. In order to identify the transcriptional interactions occurring in root tissues we used the microarray data as input for the ARACNe algorithm [16].

ARACNe is an information-theoretical method for identifying transcriptional interactions between gene products using microarray expression profile data, which is able to recover non-linear statistical dependencies between variables and has been previously used for TRN reconstruction [17-20]. In this work we show that our database, and the TRNs derived from it, have been able to recover functions and target genes for previously characterized TFs. We further show that the inferred TRNs can accurately predict new TF functions, as exemplified by the predicted role of the MADS-box TF *XAL1*/*AGL12* (AT1G71692) in secondary cell wall formation and its confirmation with loss-of-function mutant root phenotypes for this gene.

## Results and discussion

In order to infer the TRNs underlying root development and physiological processes in Arabidopsis, we used two carefully curated datasets obtained from 656 root-specific CEL files from 56 ATH1 microarray experiments (Additional file 1). The first dataset, that we call the TFs-only dataset, is a 656 columns by 2088 rows table that corresponds to our list of 2088 TF probesets. The second dataset, that we call the complete dataset, is a 656 by 22810 table that contains all 22810 probesets present in the ATH1 chip. We used both datasets as input for the ARACNe software [21]. The ARACNe output is a list of interacting probeset pairs ranked through a Mutual Information value and its associated p-value. Details for the theoretical background and practical use of ARACNe can be found in [16] and [21] but, briefly, an interaction between gene A and gene B means that the expression profile of gene A along all 656 experiments explains the expression profile of gene B along those same 656 experiments, and vice versa, as the interactions are not directed. In a biological context, an interaction between gene A and gene B will imply that gene A and gene B participate in the same physiological process and, even further, if gene A is a TF and gene B is a non-TF, the interaction (gene A explains gene B) will suggest that gene A is a transcriptional regulator of gene B.

Network inference was centered on the 2088 TF probesets present in the ATH1 chip and was obtained at three data processing inequality (DPI) values, 0.0, 0.1 and 0.2. DPI is a known information-theoretical property and is explained in the supplementary manual in [21]. Briefly, at DPI 0.0, when a three-node clique (triangle) is present, the interaction with the lowest mutual information will be removed, as this interaction is considered to represent an indirect interaction. At DPI values other than 0.0, three genes loops are allowed and, at DPI 1.0, no interactions are removed. A DPI value of 0.2 (which will preserve triangles if the difference between the mutual information value of its interactions is 20% or less) increases the recovery of true positive interactions while still minimizing the recovery of false positives [16]. After translation of the ARACNe output adjacency files into Cytoscape compatible tables, we obtained the corresponding TFs-only (TFsNet; Additional file 2) and complete (FullNet; Additional file 3) databases. As shown in Table 1, the number of edges increases dramatically from DPI 0.0 to DPI 0.1 to DPI 0.2. For clarity, all graphical representations of the networks in this paper are those obtained at DPI 0.0.

**Table 1:** Number of nodes and edges in the TFsNet and FullNet obtained at DPI 0.0, 0.1 and 0.2

**TFsNet**		
**DPI**	**Nodes**	**Edges**
0.0	2068	3574
0.1	2068	12049
0.2	2068	39113
**FullNet**		
**DPI**	**Nodes**	**Edges**
0.0	22553	82059
0.1	22553	706043
0.2	22553	2570778

Nodes and edges present at DPI 0.0 are part of the network obtained at DPI 0.1 and both are part of the network obtained at DPI 0.2.

### TFs participating in inferred interactions are expressed in roots

An important question regarding our networks is to determine if the TFs participating in the inferred interactions are actually being expressed in root tissues. The mas5calls function from the affy R package, used to flag microarray expression values as Present, Absent or Marginal, is an unreliable tool to determine if a gene is being expressed or not [22], specially when it involves Arabidopsis TFs [23]. Therefore, in order to determine if the TFs present in our networks are expressed in root tissues, we extracted from both the TFsNet and FullNet obtained at DPI 0.0 all TFs that participate in an interaction and we compared both lists to lists of experimentally determined root-expressed genes (see Methods). Results are presented in Table 2 and Additional file 4. Over 92% of the recovered TFs in the two types of networks have been experimentally determined to be expressed in roots. We are therefore confident that the TFs present in our datasets are indeed root TFs and the interactions that we have recovered represent true *in planta* transcriptional interactions.

**Table 2:** Number of TF from the TFsNet and FullNet whose expression has been experimentally detected in roots

**TFsNet**		
	**Number of TFs**	**% of total**
**Detected**	1791	92.8
**Not detected**	139	7.2
**Total**	1930	100.0
**FullNet**		
	**Number of TFs**	**% of total**
**Detected**	1835	92.3
**Not detected**	153	7.7
**Total**	1988	100.0

### TFs that participate in the same processes are grouped together in the TFsNet

**Figure 1:**
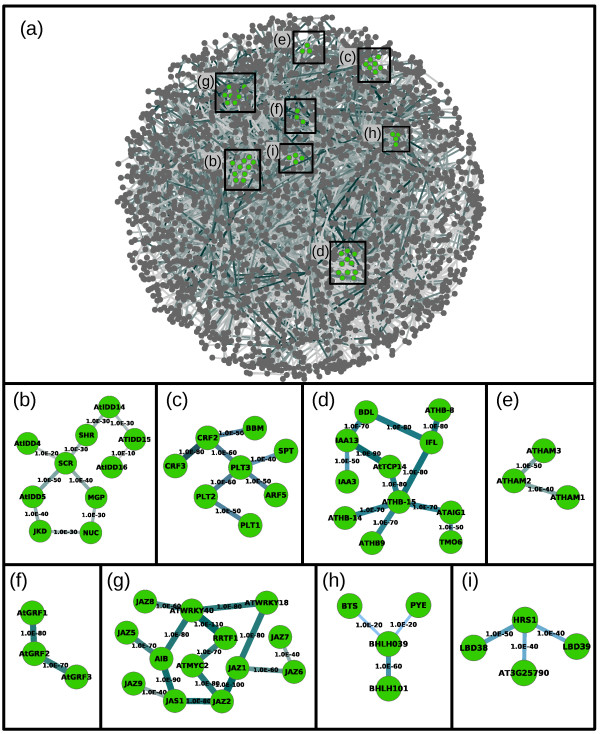
**The TFsNet. (a)** Overview of the TFsNet obtained at DPI 0.0. Genes are represented as nodes and inferred interactions as edges. Nodes are colored grey, except genes mentioned in the text that are colored green. Edge width and color intensity is proportional to the Mutual Information (MI) value of the interaction, with higher MI values corresponding to thicker and darker edges. Gene names were omitted for clarity. Zooms on particular TF groups are presented in the subsequent panels. **(b-i)** Sub-networks of TFs present in the SHR-SCR group **(b)**, the PLT group **(c)**, the vascular development group **(d)**, the AtGRF group **(e)**, the AtHAM group **(f)**, the jasmonate response group **(g)** the iron-deficiency group **(h)** and the nitrate-response group **(i)**. Edges are labeled with the p-value of the interaction. Edges from the groups to the rest of the network were omitted for clarity.

The TFsNet was obtained from a TFs-only dataset that excludes all non-TF genes and constitutes an overview of Arabidopsis roots TFs inferred interactions (Figure 1). TFs participating in the same processes are expected to be grouped together in distinct clusters or modules. Some of these functional modules have been identified and experimentally characterized and serve as probes of the reliability of the inferred networks.

Two transcriptional pathways controlling stem-cell niche patterning have been identified [24-28]. The first pathway is composed of the GRAS-family *SHORT ROOT* (*SHR*; AT4G37650) and *SCARECROW* (*SCR*; AT3G54220) and the C2H2-family, INDETERMINATE DOMAIN (IDD) *MAGPIE* (*MGP*; AT1G03840) and *JACKDAW* (*JKD*; AT5G03150). As shown in Figure 1b, these four TFs are grouped together with IDD *NUTCRACKER* (*NUC*; AT5G44160), the SSXT-domain transcriptional co-activator *ANGUSTIFOLIA 3* (*AN3*; AT5G28640) and the GRAS-family *SCARECROW-LIKE 3* (*SCL-3*; AT1G50420). *NUC* and *SCL-3* are proposed direct transcriptional targets of SHR [29-31]. Note that, as networks obtained at DPI 0.0 cannot contain triangles, the absence of an edge, for example between *SHR* and *NUC*, does not imply a lack of interaction between these two genes but merely that both genes have other interactions with better MI scores. Also, interactions between the genes in this module have relatively low MI values, corresponding to p-values of 1e-30 and 1e-40 (relative to the lowest p-value in the dataset, 1e-140). This is probably not surprising since this pathway has a complex mode of molecular interaction [32] that will hinder the ability of the ARACNe algorithm to recover their interaction from microarray data with a higher p-value [21]. Additional IDD genes, *AtIDD4* (AT2G02080), *AtIDD5* (AT2G02070), *AtIDD14* (AT1G68130), *AtIDD15* (AT2G01940) and *AtIDD16* (AT1G25250), are present in this module. Protein-protein interactions have been reported for SCL-3-NUC, MGP-SCR, MGP-SHR, MGP-JKD, SCR-JKD, and SHR-JKD [33]. On the other hand, the IDD proteins JKD and MGP regulate SHR and SCR expression and movement across root tissues via both transcriptional and protein-protein interactions [27,34]. Finally, movement of the SHR protein is abolished by the substitution of a single threonine residue in its VHIID motif, which is proposed to mediate protein-protein interactions of SHR [35] and its nuclear localization [34]. It is therefore interesting to speculate that *AtIDD4*, *AtIDD5*, *AtIDD14*, *AtIDD15* and *AtIDD16* could also be involved in root development and patterning via transcriptional regulation of, or protein-protein interactions with SHR and SCR.

The second pathway involves auxin signaling through the activation of as yet unidentified Auxin Response Factors (ARFs) and the *PLETHORA* (*PLT*) TFs, of the AP2-EREBP family. The PLT genes, *PLT1* (AT3G20840), *PLT2* (AT1G51190), *PLT3*/*AIL6* (AT5G10510) and *BABY BOOM* (*BBM*; AT5G17430)*,* have overlapping expression profiles and act in a redundant manner [26]. In the TFsNet, the four *PLT* genes are part of the same group, that also includes the bHLH *SPATULA* (AT4G36930), *ARF5*/*MONOPTEROS* (AT1G19850) and the ERF-family Cytokinin Response Factors *CRF2*/*TMO3* (AT4G23750) and *CRF3* (AT5G53290; Figure 1c). Remarkably, the four *PLETHORA* proteins [26] and *ARF5*[36] are all expressed in the seedling root stele initials. Root vascular patterning has been shown to be dependent on an auxin-cytokinin cross-talk [37] and the participation in this cross-talk of a few genes, such as *SHY2*[38], *BRX*[39] or *AHP6*[40,41] has been demonstrated. However, a transcriptional network linking the *PLETHORA* pathway and cytokinin responsive TFs is still missing. The presence of two CRF TFs in this module provides new clues in this direction.

*BODENLOS* (*BDL*; AT1G04550), a member of the Aux/IAA family, is a transcriptional inhibitor of *ARF5* and its expression is controlled by ARF5 in embryos [42]. Curiously, *BDL*, as well as two other *TARGET OF MONOPTEROS* (*TMO*) genes, *ATAIG1*/*TMO5* (AT3G25710) and *TMO6* (AT5G60200), do not group with *ARF5* in the TFsNet. Instead, they are part of a group of TFs involved in vascular development that includes genes such as *IAA13* (AT2G33310), *IAA3*/*SHY2* (AT1G04240) [39], *ATHB-14*/*PHABULOSA* (AT2G34710), *ATHB-15*/*CORONA* (AT1G52150) [43], *IFL*/*REVOLUTA* (AT5G60690) [44], *ATHB-8* (AT4G32880) [45], *ATHB9/PHAVOLUTA* (AT1G30490) and *AtTCP14* (AT3G47620) [46] (Figure 1d). *pBDL::GFP* expression has been observed in the root stele of 4–5 days-old seedlings (see Figure S6 in [42]), thus pointing to possible novel roles for these auxin-related genes in vascular development.

Other TFs involved in organ development are also grouped together in the TFsNet. For example, the closely related *ATHAM1* (AT2G45160), *ATHAM2* (AT3G60630) and *ATHAM3* (AT4G00150) genes, belonging to the GRAS family, are involved in the maintenance of meristem indeterminacy, and are functionally redundant [47,48]. These three TFs also group in the same module in the TFsNet that we inferred (Figure 1e). Another example concerns the *AtGRF* genes, of the GRF family, which are expressed in developing tissues, such as shoot tips, flower buds and roots. Single mutants of the *AtGRF1* (AT2G22840), *AtGRF2* (AT4G37740) or *AtGRF3* (AT2G36400) genes have no phenotype and double mutants have minor phenotypes [49], suggesting that these three genes have redundant roles. *AtGRF1*, *AtGRF2* and *AtGRF3* group together in the TFsNet put forward here (Figure 1f). Interestingly, our network inference also recovers the interactions *AtGRF3*-*AN3* (p-value 1e-70) and *AN3*-*SCR* (p-value 1e-40), suggesting a link between the *AtGRF* module and the *SHR*-*SCR* module during root development.

The TFsNet also recovers transcriptional interactions between genes known to participate in root physiological processes other than development. A first example concerns genes involved in jasmonate response (Figure 1g). This group includes the TIFY domain genes *JAZ1* (AT1G19180), *JAZ2* (AT1G74950), *JAZ5* (AT1G17380), *JAZ6* (AT1G72450), *JAZ7* (AT2G34600), *JAZ8* (AT1G30135), *JAZ9* (AT1G70700), *JAS1*/*JAZ10* (AT5G13220), two WRKY genes involved in pathogen response, *WRKY18* (AT4G31800) and *WRKY40* (AT1G80840) [50], the bHLH-family *AIB* (AT2G46510) [51] and *MYC2* (AT1G32640) [52] and the AP2/ERF *RRTF1* (AT4G34410). Interestingly, chromatin immunoprecipitation experiments have shown that WRKY40 binds *JAZ8* and *RRTF1* regulatory regions [53], while *MYC2* was recently shown to be involved in jasmonate-dependent root development inhibition [54].

A second example includes the bHLH TF *BHLH038* (AT3G56970), *BHLH039* (AT3G56980), *BHLH100* (AT2G41240), *BHLH101* (AT5G04150), *POPEYE* (*PYE*; AT3G47640) and the DNA-binding protein-coding *BRUTUS* (*BTS*; AT3G18290), which are involved in iron deficiency stress regulation [55,56]. *BHLH039*, *BHLH101*, *PYE* and *BTS* are grouped together in the TFsNet (Figure 1h; BHLH038 and BHLH100 are not represented in the ATH1 chip).

A third example involves nitrate response TFs [57]. The earliest TFs to be expressed in response to nitrate stimulus are *HRS1* (AT1G13300), *LBD37* (AT5G67420), *LDB38* (AT3G49940), *LBD39* (AT4G37540) and *AT3G25790* (cluster 1 in [57]). Four of these five TFs, *HRS1*, *LDB38*, *LBD39* and *AT3G25790* are grouped together in the TFsNet (Figure 1h). Note that the microarray data for Long et al., E-GEOD-21443, and Krouk et al., E-GEOD-20044 in the EBI database, were released a few days after our microarray experiments download and are not part of the data used for our analysis.

### Using the FullNet to integrate and analyze high-throughput functional genomics data

The FullNet was obtained from data which included all 22810 probesets present in the ATH1 chip, and was centered on the 2088 TF probesets list (Additional file 3). In this network, TFs will be central nodes, with their interactors, either TFs or non-TFs, as neighboring nodes. Genes participating in the same processes should again be grouped together. For example, the TF groups identified in the TFsNet are still present in the same groups in the FullNet. One must bear in mind that, in this network, non-TF nodes are present. When a non-TF interacts with two TFs, and these interactions have better MI scores than the TF-TF interaction, then the latter interaction will, at DPI 0.0, be considered an indirect interaction, and thus will not appear in the network. However, this does not mean that the TF-TF interaction does not exist, only that it is “masked” by an intermediary non-TF node. When the TF-TF MI value is not the lowest in a triangle it is visible in the DPI 0.0 FullNet. This is the case for the interactions between *PLT1*, *PLT2* and *PLT3*/*AIL6*, at p-values of 1e-50, the *SCR*-*SHR* interaction at a p-value of 1e-30, the interaction of the early nitrate-responsive TF *HRS1* with *LBD38*, *LDB39* and *AT3G25790* at p-values of 1e-40 and lower, as well as the interaction of *BHLH039* with *BHLH101* at a p-value of 1e-60 and with *PYE* at a p-value of 1e-20. Interaction between *AGL71* and *AGL72*, which was present at a p-value of 1e-20 in the TFsNet, is now recovered with a p-value of 1e-50. These two MADS-box genes have recently been shown to act redundantly in apical and axillary meristems [58].

In the FullNet, interactors of a TF node are potential target genes for that TF. If this is the case, one would expect a significant number of experimentally identified target genes for that TF to be present in the corresponding lists of ARACNe interactors. One example of a TF for which ARACNe-inferred interactions are confirmed experimentally corresponds to VND7/ANAC030 (AT1G71930). VND7 is a NAC-family TF involved in secondary cell wall synthesis and several lists of its putative target genes are available [59-62]. We compared these lists of experimentally identified *VND7* target genes with our list of *VND7* interactors from the complete dataset at DPI 0.0, 0.1 and 0.2 (Table 3 and Additional file 5). 14 out of 16 genes at DPI 0.0, 24 out of 44 at DPI 0.1 and 24 out of 107 at DPI 0.2 from our *VND7* neighbor list are differentially expressed in at least one of the experimental settings. Almost all differentially expressed genes are found at high MI values, corresponding to p-values of 1e-50 and lower. Finally, three of the four differentially expressed TFs identified by Yamaguchi et al. [62], *JLO* (AT4G00220), *MYB46* (AT5G12870) and *MYB103* (AT1G63910), are part of the *VND7* cluster in the TFsNet, at p-values of 1e-50 and lower. Curiously, a top-ranked *VND7* interactor in our dataset, the pinoresinol reductase *ATPRR1* (AT1G32100), is not present in any of the experimental *VND7* target genes lists. *ATPRR1* has, at DPI 0.0, TF interactors with higher MI values than *VND7*, suggesting that it could instead be regulated by one, or more, of these higher-score TFs. Alternatively, the *VND7*-*ATPRR1* transcriptional interaction could be age-specific and not detectable in any of the above-mentioned experimental settings.

**Table 3:** **List of *ANAC030*/*VND7 *** **interactors in the FullNet obtained at DPI 0.0**

**Locus**	**Symbol**	**Short description**	**MI**	**p-value**	**Target gene**
AT3G62160		HXXXD-type acyl-transferase family protein	0.421448	1e-70	yes^a^
AT5G60720		Protein of unknown function, DUF547	0.376219	1e-60	yes^abcd^
AT1G32100	ATPRR1	Pinoresinol reductase 1	0.375341	1e-60	no
AT1G01900	ATSBT1.1	Subtilase family protein	0.375002	1e-60	yes^bcd^
AT1G54790		GDSL-like lipase/acylhydrolase superfamily protein	0.364787	1e-60	yes^abcd^
AT2G04850		Auxin-responsive family protein	0.363144	1e-60	yes^acd^
AT3G27200		Cupredoxin superfamily protein	0.362569	1e-60	yes^abcd^
AT2G27740		Family of unknown function, DUF662	0.328449	1e-50	yes^c^
AT5G01930		Glycoside hydrolase, family 5	0.324593	1e-50	yes^acd^
AT1G47410		Unknown protein	0.293837	1e-50	yes^c^
AT5G59845		Gibberellin-regulated family protein	0.27815	1e-50	yes^ac^
AT5G38610		Plant invertase/pectin methylesterase inhibitor superfamily protein	0.27147	1e-40	yes^abcd^
AT5G26330		Cupredoxin superfamily protein	0.252517	1e-40	yes^a^
AT1G24600		Unknown protein	0.243778	1e-40	yes^acd^
AT4G39320		Microtubule-associated protein-related	0.188269	1e-30	yes^c^
AT2G31085	CLE6	CLAVATA3/ESR-RELATED 6	0.180814	1e-30	no

MI: mutual information value.

The target gene column indicates if the gene is an experimentally identified target gene in [60]^(a)^, [61]^(b)^, an upregulated gene in [62]^(c)^ or a putative direct target gene in [62]^(d)^.

There are also examples of TFs for which there is little overlap between ARACNe-inferred interactors lists and experimental target gene lists. Two examples are the SHR and SCR TFs. *SHR* and *SCR* are important genes for root development and several lists of their proposed transcriptional target genes are available [29-31,63]. Sozzani et al. [30] obtained, through microarray data analysis, a comprehensive list of differentially expressed genes during a time-course of SCR or SHR induction, while Cui et al. [31] identified SHR target genes through chromatin inmunoprecipitation (ChIP). A direct comparison of the target gene lists from Sozzani et al., to which we will refer as the Sozzani-SCR and Sozzani-SHR lists, to our ARACNe list of inferred SCR or SHR interactors obtained at DPI 0.0, 0.1 and 0.2, resulted in a low overall overlap: there are 732 ARACNe-SCR and 719 ARACNe-SHR interactors at DPI 0.2, of which 68 (9.2%) and 159 (22%) were found in the corresponding SCR- or SHR-Sozzani lists. In particular, we would expect to find in both the ARACNe and Sozzani lists genes known to participate in the SHR-SCR transcriptional regulation pathway, namely *JKD*, *MGP*, *NUC* and *CYCD6;1* (AT4G03270). The first three genes are TFs and they can be found in the same module as *SHR* and *SCR* in the TFsNet. *CYCD6;1*, a non-TF, is present in both the SCR-Sozzani and SHR-Sozzani lists, but is not an ARACNE-inferred interactor of *SHR*, *SCR*, *JKD*, *MGP* nor *NUC*. At DPI 0.0 its only interacting TF is *AGL92* (AT1G31640), which is not close to the *SHR*-*SCR* module in either the TFsNet or FullNet. While disappointing, this result is perhaps not surprising: CYCD6;1 is expressed in very particular wild type root cell types, the cortex/endodermis initial stem cells and lateral root primordium endodermal cells [30,64]. Furthermore, CYCD6;1 participates in a complex regulatory mechanism involving protein-protein interactions, protein phosphorylation and protein degradation [64]. It is likely that these mechanisms are poorly translated into transcript levels of the corresponding genes in whole root samples, which is the input data for ARACNe.

**Figure 2:**
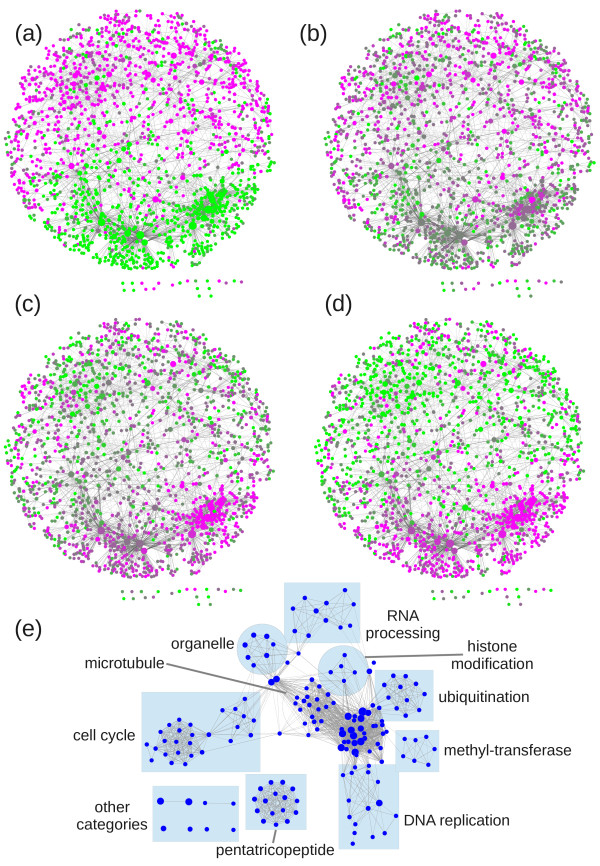
**Subnetwork of differentially expressed genes in a SHR induction time-course **[30]**.** Node colors correspond to down-regulated (green) or up-regulated (magenta) genes at 1 hour **(a)**, 3 hours **(b)**, 6 hours **(c)** and 12 hours **(d)** after SHR induction. **(e)** Enrichment Map [72] network of category enrichments calculated with David [70,71] for genes in the subnetwork that switch from a down-regulated to an up-regulated status during the induction kinetic.

The ability of ARACNe to recover experimentally identified TF target genes will most likely mirror the number and complexity of the regulatory interactions in which that TF participates. VND7 is a TF involved exclusively in secondary cell-wall synthesis (SCWS) [65,66]. As such, we expect VND7 to participate in a very specific transcriptional module, and ARACNe to accurately recover its experimentally identified target genes. On the other hand, SHR and SCR are most likely involved in numerous transcriptional pathways, as mutants for these genes are strongly affected in root development [67,68], and over 200 TFs can be found in the lists of differentially expressed genes for SHR or SCR inductions, which analyzed a specific root cell-type, i.e. ground tissue [30]. Such an important number of differentially expressed TFs (approximately 10% of all Arabidopsis TFs) further suggests that a significant number of these experimentally identified target genes are indirect targets. Additionally, regulation of root development by SCR and SHR involves expression in defined cell types, transport across cell-types, nucleus-cytoplasm translocation, protein-protein interactions and protein phosphorylation [27,30,34,35,64]. In this case, we expect that better results could be obtained by visualizing experimentally identified target genes in the context of the networks where they participate. We therefore decided to retrieve from the FullNet dataset, obtained at DPI 0.0 and with a cutoff p-value of 1e-30, all interactions for which both nodes are present in the list of 2481 differentially expressed genes in the SHR induction kinetic from Sozzani and collaborator’s study [30], to which we added *SHR* (AT4G37650). The resulting dataset now contains 1668 genes (67% of the original list) and the corresponding network was drawn with Cytoscape [69]. 1647 nodes (66%), including *SHR*, are grouped together in a single subnetwork (Figures 2a-d). We observe that this subnetwork is clearly divided in two sections, corresponding to genes that, as time progresses in the induction kinetic, switch from an under-expressed to an over-expressed state and vice versa. An analysis of this subnetwork can now help identify relevant nodes, which should play important roles in the SHR transcriptional pathway. For example, three of the main nodes that switch from under- to over-expression are *PRMT3* (AT3G12270), *KYP* (AT5G13960) and *HD2A* (AT3G44750), which are genes coding for chromatin modification (histone methyl-transferase and histone deacetylase) proteins. An analysis of all genes that switch from under- to over-expression when using David [70,71] and Enrichment Map [72] reveals that this module is enriched, among others, in cell-cycle, microtubule, RNA-processing and putative chromatin modification protein-coding genes (Figure 2e).

### ARACNe-inferred networks allow for the prediction of novel genetic interactions for root-expressed TFs: a possible role for *SPATULA* in the *PLETHORA* pathway

**Figure 3:**
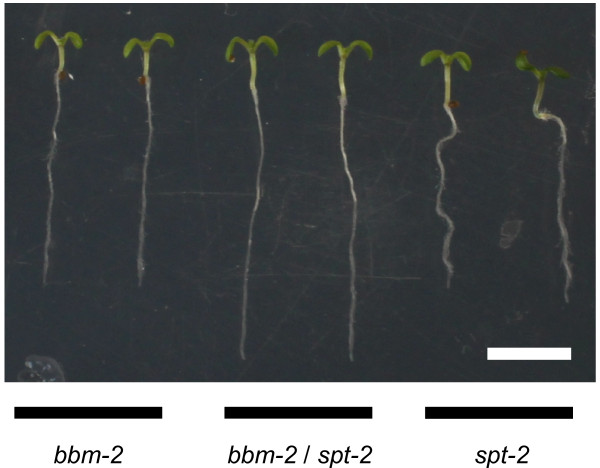
**Photographs of *bbm-2*, *spt-2 *and *bbm-2*/*spt-2 *six days-old mutant seedlings grown on vertical plates.** Bar represents 0.5 cm.

The TFsNet was obtained from data which included exclusively our list of 2088 TF probesets (see Methods). In this network, TFs that participate in the same biological process should be grouped together. Therefore, we expect higher order mutant plants for genes in a same module to exhibit root phenotypes not observed in single mutant plants. We set to test this hypothesis with genes that are present in the same module, but 1) belong to different TF families, 2) are not immediate neighbors in the TFsNet, and 3) whose mutants have distinct root phenotypes. The genes *BABY BOOM* (*BBM*) and *SPATULA* (*SPT*) matched these criteria. Both genes are present in the same module (Figure 1c), and mutants of the *BBM* gene, an AP2-domain TF, have slightly shorter roots [26], while mutants of the *SPT* gene, a bHLH TF, have slightly longer roots than wild type plants [73]. When grown on vertical plates, the *bbm-2*/*spt-2* double mutant exhibited longer roots than either *spt-2* or *bbm-2* single mutant seedlings (Figure 3). A previous report showed that *PIN4* and *DR5::GUS* expression is altered in the root meristem of *spt-11* mutant seedlings [73]. Taken together, these results point to a possible transcriptional interaction between the *PLETHORA* pathway and *SPATULA* in the regulation of auxin transport and/or response in Arabidopsis root meristems.

### ARACNe-inferred networks allow for the prediction of novel functions for root-expressed TFs: the case of *XAL1*/*AGL12*, a MADS-box TF involved in secondary cell-wall synthesis

Since our ARACNe inferred networks are able to recover known gene associations, we expect them to also be able to predict novel TF functions. As an example of the predictive power of our database, we decided to look for new TFs that could be participating in secondary cell wall synthesis (SCWS). For this aim, our strategy consisted in selecting several genes, both TF and non-TF, known to be involved in SCWS, recover their interactions from the FullNet and draw the resulting network in order to identify new SCWS TFs. Several TFs are known to be involved in SCWS, among which we chose *VND6*/*ANAC101* (AT5G62380), *VND7*/*ANAC030* (AT1G71930) [74], *SND2*/*ANAC073* (AT4G28500) [75], *MYB46*[76] and *IXR11* (AT1G62990) [77]. As SCWS non-TF genes we chose the cellulose synthases *CESA4* (AT5G44030), *CESA7* (AT5G17420) and *CESA8* (AT4G18780) [78], the laccases *LAC4* (AT2G38080) and *LAC17* (AT5G60020) [79], the cysteine peptidases *XCP1* (AT4G35350) and *XCP2* (AT1G20850) [80], the chitinase-like *ATCTL2* (AT3G16920) [81], the DUF6 domain *WAT1* (AT1G75500) [82], *TED6* (AT1G43790) [83], the DUF231 domain *TBL3* (AT5G01360) [84] and the family 8 glycosyl-transferase *GAUT12*/*IRX8* (AT5G54690) [85]. We then retrieved from the FullNet all interactions involving these genes at DPI 0.0 and a p-value cutoff of 1e-30 and used Cytoscape [69] to visualize the corresponding network (Additional file 6). It immediately appears that these genes are indeed part of a network of SCWS genes that includes our input genes plus several other known, or putative, SCWS genes including *MYB83* (AT3G08500) [86], *ANAC007*/*VND4* (AT1G12260) [65] or *ATPRR1*[87], but also vascular development TFs like *ATHB-15*[43], *ATHB-16* (AT4G40060) [88] and *JLO* (AT4G00220) a target of VND7 [62].

**Figure 4:**
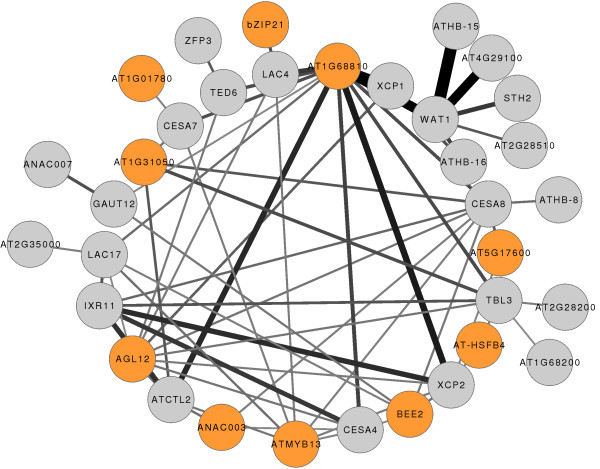
**Highly connected part of the SCWS network obtained at DPI 0.0 and a p-value cutoff of 1e-30.** Genes are represented as nodes and inferred interactions as edges. TFs not present in the input list are colored yellow. The TFs AT1G68810, BEE2 and AGL12 (XAL1) are further mentioned in the text and colored orange. Edge width is proportional to the Mutual Information (MI) value of the interaction, with higher MI values corresponding to thicker edges.

In a highly connected part of this SCWS network, 22 TFs that were not part of our input gene list are now present (Figure 4). We retrieved from the FullNet all interactions involving these TFs at DPI 0.0, 0.1 and 0.2 and a p-value cutoff of 1e-30. An enrichment analysis, using David, of the lists of interactors for three of the newly identified TFs, *XAL1*/*AGL12* (a MADS-box), *BEE2* and *AT1G68810* (two bHLH) revealed that they are particularly enriched in SCWS genes (data not shown); the lists of high MI value interactors for each TF are shown in Additional file 7. As these three TFs are present in the highly connected part of the SCWS network, it is not surprising to find that they share several of their interactors. *AGL12*/*XAL1* is a MADS-box transcription factor that is expressed in phloem tissues and is involved in the regulation of both root development and flowering time [89]. *BEE2* was first identified as a brassinosteroid-responsive TF [90]. Brassinosteroids promote root growth [91], are essential for the development of the vascular system in Arabidopsis stems [92] and enhance xylem vessel transdifferentiation of Arabidopsis suspension cultures [74]. *AT1G68810* is 1) a TF that we found as part of the vascular development cluster in the TFsNet, 2) closely related to *ATAIG1*/*TMO5,* which is also part of the TFs-only vascular development cluster and 3) a protein-protein interactor of LONESOME HIGHWAY, a transcriptional activator involved in vascular development [93]. These results predict that *XAL1*, *BEE2* and *AT1G68810* are important TFs for SCWS.

**Figure 5:**
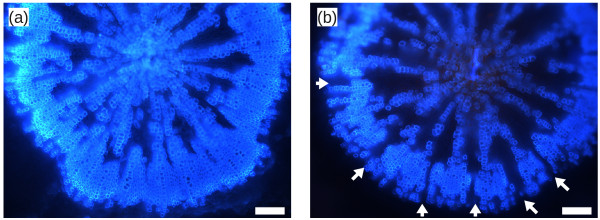
**Photographs of wild type (a) and *xal1-2 *(b) adult root transverse sections observed under UV light.** Autofluorescence of lignified tissues is evidenced as a light-blue coloration. White arrows in *xal1-2* indicate gaps in the xylem-fiber ring. Bars represent 100 microns.

As MADS-box TFs are not usually associated with SCWS, we decided to look for SCW deposition in *xal1-2* loss-of-function mutant roots [89]. Since *xal1-2* presents a delay in flowering time, roots from plants of the same chronological age might reveal developmental stage-related SCWS differences rather than a direct SCWS phenotype. Therefore, both Col-0 and *xal1-2* roots were collected when the main stem was 29–32 cm in length, which arguably corresponds to plants at the same developmental stage. As predicted by our inferred network, *xal1-2* adult roots indeed have altered secondary cell-wall patterns with gaps in the secondary xylem and fiber ring (n = 10/10), a phenotype rarely observed in wild type plants of the same size (n = 1/10; Figure 5). In an intriguing paper, Sibout et al. have shown that xylem expansion in hypocotyls and roots is linked to flowering time [94]. Coincidentally, *xal1-2* plants have delayed flowering [89] and altered root SCWS, strongly suggesting that *XAL1* could be part of a TRN that connects both processes.

The confirmation of SCWS alterations in *xal1-2* root tissues shows that our bioinformatics methodology to infer TRNs is a successful approach for the accurate prediction of novel functions for root-expressed TFs. This result further strengthens that our networks will likely provide novel hypothesis concerning functional modules involved in root development. As an additional example, the DUF6 protein WAT1 [82] has, at DPI 0.0 and a p-value cutoff of 1e-50, the TF interactors *ATHB-15*/*CNA*, *AT1G68810*, *AT4G29100*, *STH2*, *ATHB-16* and *AT2G28510*, all of which are part of the vascular development cluster of the TFsNet (Figure 1d). This suggests, first, that one, or more, of these TFs is the transcriptional regulator of *WAT1* in root tissues and, second, that one, or more, of these TFs control vascular development, at least partly, through the direct transcriptional regulation of *WAT1*. Finally, the DUF6-domain protein-coding genes *AT1G43650*, *AT1G01070*, *AT3G45870*, *AT3G18200* and *AT4G30420* are interactors of TFs known to be involved in SCWS, suggesting that they might have similar roles to *WAT1* in root SCWS.

## Conclusions

In this work we show that network inference from multiple compounded, carefully selected and curated microarray datasets allows for the reconstruction of reliable root transcriptional interaction networks. We show that such inferred networks recover both known, functionally characterized TF modules and reliably predict novel components of such modules, as well as novel modules, including unexpected roles for particular TFs. We particularly highlight the discovery of a new module underlying secondary cell wall synthesis that involves the MADS-box TF *XAL1*/*AGL12*. Our transcriptional interactions database further provides an overview of the transcriptional pathways present in Arabidopsis roots and will likely yield a plethora of novel hypotheses to be tested experimentally.

## Methods

### Microarray data

A list of all microarray experiments using the Affymetrix GeneChip ATH1-121501 was downloaded on October 2010 from the EBI ArrayExpress database [8] (Additional file 1). Using the corresponding sample-data relationship files as a guide, all experiments using root tissues were selected and the corresponding CEL files were retrieved. In experiments involving tissue comparisons, for example shoot vs root, particular care was taken to exclude non-root CEL files. Also, in order to obtain a high quality, homogeneous dataset, the arrayQualityMetrics Bioconductor package [95] was ran on each experiment and low quality CEL files were excluded from further analysis, as were CEL files corresponding to samples from ecotypes other than Columbia-0. Finally, in order to avoid possible perturbations of the underlying Gene Regulatory Network [96], all CEL files corresponding to transgenic samples (mutants, overexpressions, promoter constructions) were also excluded. This resulted in 656 CEL files that were normalized using gcRMA [97] under R. The resulting normalized data was used as input for the ARACNe algorithm.

For TFs-only networks, the selected root CEL files were transformed to ASCII format using the celutil utility [98] and the ATH1-121501 array name was replaced with a custom name. A TFs-only CDF file was created using a modified version of the XSpecies [99] use_ME.pl Perl script [100] and the Affymetrix ATH1-121501 probe_tab file was renamed to match the custom CDF name. Both files were packaged for R using the makecdfenv and AnnotationDbi packages, respectively, which allowed us to normalize the modified CEL files with gcRMA [97]. The resulting normalized data was used as input for the ARACNe algorithm.

### Transcription factors list

The -e or Data Processing Inequality ARACNe parameter uses a list of TF in order to preserve interactions including one or more TFs [21]. There are three Arabidopsis TF databases, Agris [1,2], RARTF [3,4] and DATF [5,6]. As DATF includes the Aux/IAA family as a TF family, while Agris and DATF do not, and since auxin is a major player in plant development, we decided to create our own TF list by combining all AGI IDs present in the three databases. We further added all AGI IDs from the TAIR10 ATH_GO_GOSLIM file that were annotated with the Gene Ontology entry GO:0003700, “sequence-specific DNA binding transcription factor activity”, as several TFs, like AGL26 or AGL64, were missing from the databases. The final TF list contains 2575 AGI IDs corresponding to 2088 probesets (Additional file 8).

### Network inference using ARACNe

Normalized data was used to calculate the config_kernel.txt and config_threshold.txt parameters required by ARACNe using the author provided Matlab scripts. Interactions were inferred for the 2088 TF probesets using the Linux command-line ARACNe 32 bit program at three DPI values, 0.0, 0.1 and 0.2, with the 2088 probeset list as the -l parameter for the complete dataset or without the -l parameter for the TF-only dataset. The command-line execution of the ARACNe program was in the form: aracne –H config_parameters -i normalized_data [-l TF-list] -e 0.0/0.1/0.2 -o output_file.

### Adjacency files transformation

The TAIR10 *array_elements* and *aliases* tables were combined in order to obtain a single table linking probesets to AGI IDs and, when available, their corresponding symbols. The resulting combined table was used to transform the ARACNe output adjacency files to Cytoscape compatible, tab-delimited tables using a custom Perl script.

### List of root-expressed TFs

Root expressed TFs were identified by combining a) the list of TFs detected in 14 days-old seedling roots by real-time RT-PCR [23], b) a list of proteins identified in large scale proteomic screens of root samples, experiments 3332, 15486, 15489, 15517, 15518, 15519, 15525, 15526, 15528 in the EBI PRIDE database [101-103], c) the list of genes annotated as being expressed in roots or whole plant in the TAIR10 Plant Ontology table, i.e. containing a Gene Ontology experimental evidence code, excluding microarray evidence, and d) a list of root expressed genes from RNA-seq experiments, accessions SRR314814 [104] and SRR331219 from the DNA Data Bank of Japan Sequence Read Archive [105]. Primers used by Czechowski et al. [23] were Blasted to the TAIR10 genome to confirm that they were still specific for the intended genes. For RNA-seq data, fastq reads were aligned to the TAIR10 cDNA sequences using bowtie2 (version 2.0.0-beta5; [106]) with the *--very-sensitive*, -*N 1*, -*k 11* and *-S,0,-0.8* parameters in end-to-end mode. Only reads matching a single locus were considered for the identification of expressed genes using a custom Perl script.

### *bbm-2*/*spt-2* double mutant

*bbm-2* and *spt-2* seeds were obtained from the Arabidopsis Biological Resource Center. Adult *bbm-2* and *spt-2* plants were crossed and homozygous double mutant plants were identified by genotyping of the *bbm-2* mutation using the *BBM* primers 5'-ACTTTAGTGCGGCTAAATCGTAAGC-3′, 5′-CAATAACGAACAAAATGGACCAAAG-3′ and LBb1.3 primer 5′-ATTTTGCCGATTTCGGAAC-3′, and by visual identification of plants exhibiting the *spt-2* split carpels phenotype. Seeds for both single mutants and the homozygous double mutant were sown on 0.5X Murashige and Skoog basal medium, 0.5% saccharose, 1% plant agar (*Phyto*Technology Laboratories) plates. Plates were placed at 4 °C, and after two days transferred to a growth chamber at 22 °C with a long day light regime (16 hours light, 8 hours dark). Photographs were taken at six days post-germination.

### Microscopy

Col-0 and *xal1-2*[89] plants were grown in soil under standard greenhouse conditions. Plants with a 29–32 cm high main stem were collected, transverse sections of the root below the hypocotyl were hand-cut and autofluorescence of the lignified tissues was immediately observed under UV light with a fluorescence microscope.

## Abbreviations

TF: Transcription factor; DPI: Data processing inequality; AGI ID: Arabidopsis Genome Initiative locus identification number.

## Competing interests

The authors declare that they have no competing interests.

## Authors’ contributions

RACM participated in the design of the study, realized all bioinformatics analysis, performed experiments and wrote the manuscript. GC participated in the design of the study and participated in data analysis. KLGA and NMM performed experiments. SdF helped to write the manuscript. ERAB conceived the study, participated in the design of the study, discussed data analyses and results, and helped to write the manuscript. All authors read and approved the final manuscript.

## Supplementary Material

Additional file 1 Table of ArrayExpress experiments used as microarray data sources for this work. Click here for file

Additional file 2 Cytoscape compatible table for the TFsNet obtained at DPI 0.0. Click here for file

Additional file 3 Cytoscape compatible table for the FullNet obtained at DPI 0.0. Click here for file

Additional file 4 Table of experimental evidence of root expression for all TFs present in the TFsNet and FullNet. Click here for file

Additional file 5 **List of *ANAC030*/*VND7 *** **inferred interactors in the FullNet obtained at DPI 0.0, 0.1 and 0.2.** Click here for file

Additional file 6 **Figure of the SCWS subnetwork obtained at DPI 0.0 and a p-value cutoff of 1e-30.** Genes are represented as nodes and inferred interactions as edges. Nodes corresponding to the input genes mentioned in the text are colored green. Edge width is proportional to the Mutual Information (MI) value of the interaction, with higher MI values corresponding to thicker edges. Click here for file

Additional file 7 **Table of *XAL1*/*AGL12*, *AT1G68810 *and *BEE2 *inferred interactors in the FullNet obtained at DPI 0.0, 0.1 and 0.2.** Only interactors with a p-value of 1e-50 or less are shown. Click here for file

Additional file 8 List of Arabidopsis loci considered as TFs in this work. Click here for file
