# Supplementary figures and images for: ARACNe-based inference, using curated microarray data, of Arabidopsis thaliana root transcriptional regulatory networks
Source: BMC Plant Biol. 2014 Apr 16;14:97. doi: 10.1186/1471-2229-14-97 (PMC4021103; doi:10.1186/1471-2229-14-97)

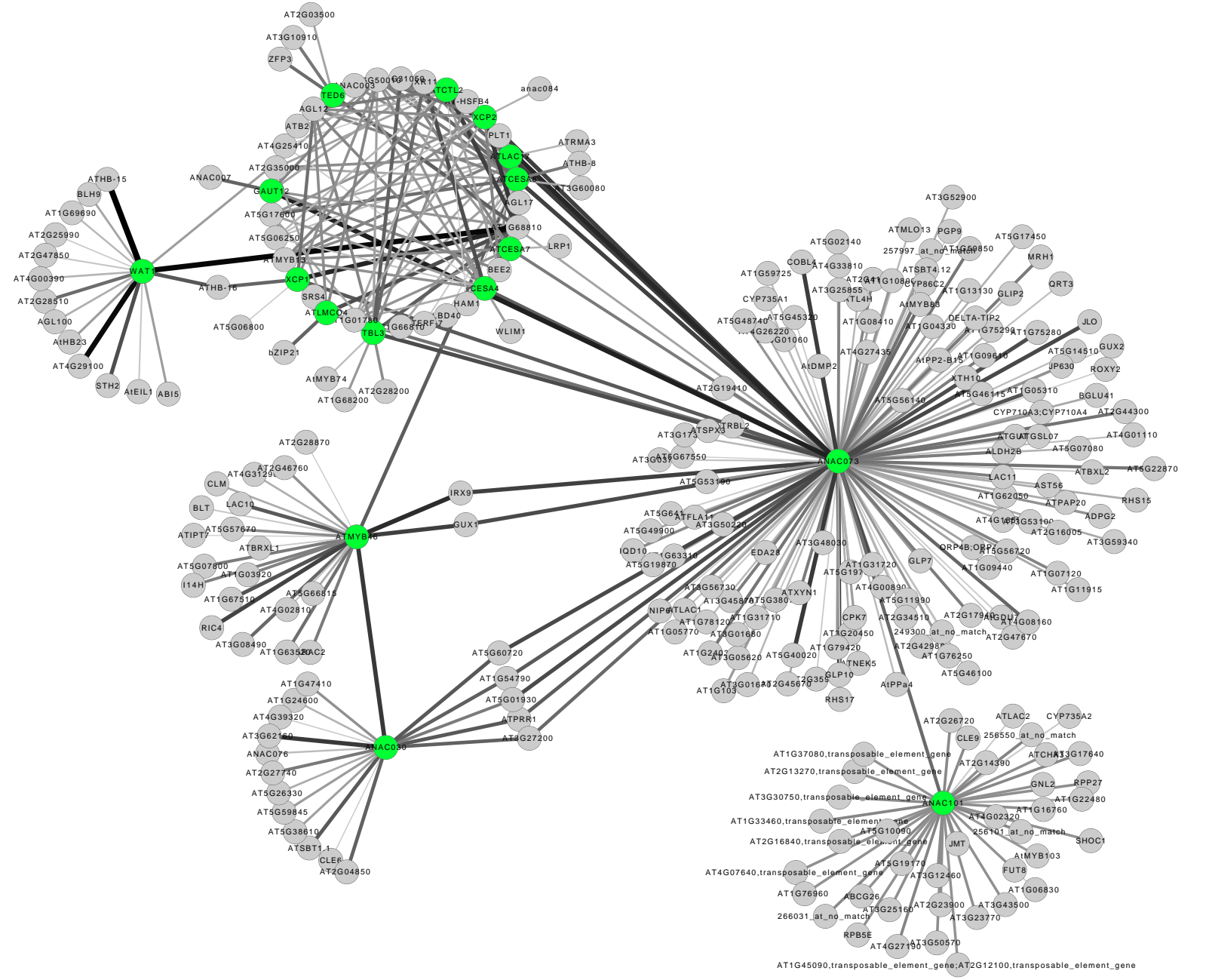

Supplement: Additional file 6 — Figure of the SCWS subnetwork obtained at DPI 0.0 and a p-value cutoff of 1e-30. Genes are represented as nodes and inferred interactions as edges. Nodes corresponding to the input genes mentioned in the text are colored green. Edge width is proportional to the Mutual Information (MI) value of the interaction, with higher MI values corresponding to thicker edges. [file 1471-2229-14-97-S6.pdf]
